# What Shall We Do with the Uterus after Abortions?

**Published:** 1892-08

**Authors:** K. P. Moore

**Affiliations:** Macon, Ga.


ATLANTA
MEDICAL AND SURGICAL JOURNAL.

VOL. IX. AUGUST, 1892. No. 6.

EDITED BY
LUTHER B. GRANDY, M. D., AND MILLER B. HUTCHINS, M. D.,
WITH THE CO-OPERATION OF
H. V. M. MILLER, M. D., LL. D., VIRGIL O. HARDON, M. D., AND
FLOYD W. McRAE, M. D.

### ORIGINAL COMMUNICATIONS.

### WHAT SHALL WE DO WITH THE UTERUS AFTER ABORTIONS?

BY
K. P. MOORE, M. D.,
MACON, GA.

Mrs. P., aged about thirty-two, a lady from Minnesota, while in one of our Southern cities this winter, had an abortion at the third or fourth month. The third or fourth day after her abortion she was allowed to be up and on her feet. She was making a tour of the South, and hence was constantly on the go. Soon after the abortion she began to have a constant flow, sometimes more, sometimes less, and never at any time feeling well. She was visiting many of the Southern cities, and about three weeks after the abortion she chanced to strike our fair city. During all this time the flow continued to increase, her strength to fail, and she was now forced to take her bed. On March 4th, 1892, she was admitted into my private sanitarium. I found her with a temperature of 102; pulse, 130, with considerable pelvic and abdominal pain, some tympany, and nausea; great uterine tenderness, and an area of tenderness and hardness on left side of uterus. Diagnosis: Metritis, local pelvic peritonitis, with probable salpingitis. There was a muddy, offensive, sanguinolent discharge. The next morning after her admission the temperature was 103, with a corresponding aggravation of all the other symptoms.

Could a more grave condition be presented? Every one who has had to deal with peritonitis, especially of an infectious or septic origin, realizes at once the serious outlook which surrounded this patient. The etiology and pathology in this case is plain.

There remains in this uterus portions of the uterine contents of the previous conception. Decomposition of these membranes and blood clots has been the source of infection. What shall be done?

We are taught by some authorities of unquestioned ability that the uterus is an organ whose interior is too sacred to be invaded at all. Here I have a bleeding endometrium, with a septic discharge, and, under ordinary circumstances, and on general principles, perhaps the most conservative gynæcologist would arm himself with a curette and attack the enemy, although he was barricaded in this sacred fort and fortified by strong and able authority against its invasion.

But in addition to this condition of the *uterus*, we have here a peritonitis, which should be gently coaxed into submission by the most tender persuasion. Would not pulling on this uterus, dilating its cervical canal, curetting its interior, and otherwise roughly handling this sacred organ, fire up afresh the local peritonitis, and make great risk of its becoming a general peritonitis? My anxious solicitude can well be imagined.

Do you ask what all this has to do with the management of the uterus after abortion? I answer, much every way. The precarious condition of this patient had its origin in a failure to do for her what should have been done at the time of her abortion, and if she should have died, to a certain degree, her death would, in my humble judgment, have been chargeable to a failure on the part of her physician to do his *whole* duty.

In the early months of gestation the large mass of deciduous membrane is apt to be retained after the ovum has escaped from the uterus. Even if the bulk of the mass has passed out, its firm attachment to the uterine walls renders the retention of portions of this mass quite probable. After the formation of the placenta, portions of this organ is also liable to be left, and these undetached membranes and tissues not only afford sources of sometimes constant and dangerous hemorrhages, but on account of their own decomposition, as well as the decomposition of retained blood clots, become a fruitful soil for the propagation of germs, and the inauguration of a septic condition hazardous to the life of the patient.

Two plans for the management of the uterus after abortions are recommended, and each have advocates of high and unquestioned ability.

One, I might designate as the active or aggressive plan, the the other the conservative or expectant plan. I prefer to call it expectant rather than conservative, for my idea of conservatism is not merely to stand by and see a patient get well, and trust alone to *vis medicatrix naturæ*, but true conservatism consists in active interference when necessary, and conserving the vital forces, as far as skill and art, directed by sound judgment, can do it.

I would not be understood as discounting the opinions of those who advocate the expectant plan, and who hold that we should leave the matter to nature until a subsequent hemorrhage or an odorous discharge should afford an indication for active interference.

Tarnier advises non-interference even if the whole placenta is known to be in the uterus. He insists that the uterus should be allowed time to expel its contents by a due process of nature, but in the meantime he urges systematic antiseptic injections, and yields only to active interference when alarming hemorrhage or a foul odor presents the indications for interference. In

France the burden of authority is rather in favor of the expectant course.

Tarnier refers to the statistics of the Charité and Maternité where he saw forty-six cases of retained placenta, with only one death, and that from pneumonia, but in the hospital of Florence, where the same course is pursued, the statistics show a death rate of six per cent. In Germany, while there is great division of opinion, the preponderance of testimony is in favor of active interference. Schroeder says the abortion must not be hastened until the os is fairly dilated and the ovum well separated from the uterus, but, says this eminent authority, if any portion should remain behind it must be invariably removed, even should the cervix have to be split on both sides to reach it. Fehling and Schwarz are also warm advocates of the active plan. Braun deprecates the early use of instruments, but advises the use of the finger whenever possible to remove the ovum. Dohrn recommends the expectant plan to the farthest limits, and Winckel advises no active interference.

In our country difference of opinion exists among men preeminent in their respective spheres. Such men as Munde, Polk, Wylie and others are decided in their teachings as to the duty and importance of active measures, while Parvin, Thomas and other leaders, whose opinions are always entitled to the highest consideration, are on the other side of the question. Hirst, in the *American System of Obstetrics*, and to whose article I am largely indebted for the opinions of authors quoted, laconically presents the question in this way: "Is the retention of decidua, fœtal membranes or placenta after abortions fraught with any danger to the woman? And is the immediate removal of the secundines after abortion necessarily a violent or dangerous procedure?"

In the decision of many matters in the practice of medicine we are to make a choice of two evils, and it seems to me that we might present the question in this way: Which plan would be the most risky to the patient, curetting and leaving the womb in a clean, well drained condition, or take the chances of leaving decidual or fœtal remains in the uterus and trust to the physiological processes of nature to rid herself of them? The answer to the question will depend largely upon the *method* pursued. If the active course is *properly* done, there is no question in my mind that it is our duty, under all the circumstances, to leave the uterus entirely clean and free from any possible risk of subsequent infection from decomposing material. If, on the other hand, it is *not properly* done, then, perhaps, safety lies on the line of expectancy.

Duhrssen has recently reported one hundred and fifty cases of abortion treated by an immediate and thorough cleaning out of the uterine cavity, with only two deaths and these in no manner attributable to the treatment adopted. His proposition is to "treat the ovum before the third month of pregnancy like a polypoid tumor, and so soon as the os is slightly dilated to introduce a curette and incontinently clear the uterus of its contents." Perhaps this is an extreme position, and I can see nothing to be lost by allowing a reasonable time for the uterus to expel its contents, and then curette.

Hirst says that after the ovum is wholly or in part expelled everything left behind in the uterine cavity, whether thickened decidua or placental tissue, is to be extracted. This strikes me as the true conservative course.

How shall this be done? Hirst recommends as the best instrument, especially in retained placental portions, the finger of the physician, claiming that in this way the attached portions can be recognized and peeled off from the uterine walls and easily extracted. In this I take issue with this eminent authority. Who of us have not wrestled in vain to get away those attached portions when the uterus was out of reach? and with much difficulty the finger was barely introduced, and though the sense of touch acquainted us with the presence of foreign remains, yet we found it impossible to manipulate the finger sufficiently to detach them. Besides this it is not possible to do the operation with the same degree of cleanliness and as aseptically with the finger as with the curette. The curette, speculum, depressor, tenaculum forceps, and every necessary instrument can be made absolutely aseptic by boiling, which could not be done with the finger. Again, however careful we may be in cleaning the vulva and vagina, there would be some probability of conveying germs into the uterus on even a thoroughly cleansed finger, which need not be the case with the curette, for the curette need never touch the vaginal walls or external genitals at all.

How should the operation be done? It is an operation, and should be so regarded whenever attempted. Not only should it be regarded as an operation but as one involving vital issues, and entitled to as much care and precaution as a laparotomy or any other capital operation; and when these precautions are taken, in my judgment the records will show 100 per cent. of recoveries.

It has been the custom of myself and excellent co-partner for some time now to pursue the *active* plan in every case of abortion that has come into our hands, and though we cannot as yet present a long list of statistics, yet we have had quite a number, and the results have been so satisfactory that we would now al-almost feel ourselves criminal to neglect the use of this plan.

The simplicity of the plan and the meagre supply of instruments actually needed places the operation within the reach of every practitioner. The necessary instruments are a Sims speculum (I prefer a Munde's or Taliaferro's modification of Sims), a dull curette, a uterine or vaginal depressor, a pair of applicators or uterine forceps, and a pair of tenaculum forceps. These should all be put into boiling water for ten or fifteen minutes before use; this secures thorough sterilization. The vulva, mons and insides of the thighs should now be made absolutely clean with soap and water; also thoroughly irrigate the vagina with the same. Now wash the genitals and vagina with warm solution of bichloride-mercury about 1-3000. Now place the patient in an exaggerated Sims position with the hips close to the edge of the table or bed (we have always used the bed). Through the speculum we now place a large pledget of absorbent cotton, which has been soaked in the mercuric solution, against the cervix and allow it to remain here to take up any oozing, as well as to guard the cavity until other preparations are completed.

We now prepare a strip of moist iodoform gauze about one inch wide, and three feet long for filling the uterine cavity, and another strip six inches wide for the vaginal cavity. These can be dropped into hot water or the mercurial solution if deemed advisable, but if the gauze has been well kept in a sealed can, and is quite moist, I do not feel that it is necessary to further sterilize it. We are now ready for curetting. Perhaps as a general rule it would be well to use an anæsthetic, as it not only saves pain and economizes nerve force, but it places the patient entirely in your hands without resistance; although it is not a necessity to use the anæsthesia, as we find most our patients prefer not to take it. Now remove the cotton from the vagina, and with the tenaculum forceps gently draw down and steady the uterus, while its entire cavity is thoroughly curetted. If the cervical canal is not large enough to admit of perfect freedom in the use of the curette, and to secure free drainage, it should be well dilated before commencing to curette. Now irrigate the uterus with warm sterilized salt water, and wash out all the débris. An ordinary soft rubber catheter attached to a pump or fountain syringe will answer every purpose. Of course there should be plenty of room for the reflow by the side of the catheter. Now that the uterus is cleared of its contents, and well washed out, sprinkle the narrow strip of iodoform gauze with tr. iodine, or Lugol's solution, and pack the uterine cavity with it, leaving the free end in the vagina to facilitate its removal. We now sponge out the vaginal canal with bichloride solution and loosely fill the vagina with the wide strip of gauze. This completes the operation.

The dressing is allowed to remain twenty-four to forty-eight hours, or even to seventy-two hours. I have never found the slightest odor after removing the dressing, and have never had a case that did not go on to a speedy recovery with never a single unpleasant result. I now direct a warm salt vaginal douche every day for a week or more, although there is usually but little discharge, and involution goes on rapidly and healthfully, and I have no fear of subsequent hemorrhage, subinvolution or chronic metritis.

The drainage is one of the fundamental principles of successful surgery, and is, if possible, more applicable to gynecological than general surgery.

In the case reported in the beginning of this paper, I promptly gave the uterus a thorough curetting and irrigation, and in ten hours time the temperature fell from 103 to below one hundred, and she went on to a complete recovery, and in seven weeks was speeding on her journey to her far-away Western home. In curetting this uterus I incurred the risk of firing up the local peritonitis into a general conflagration of the whole peritoneal cavity, which, as we all know, would mean death to my patient, yet what hope was there in leaving this smouldering source of infection, which had already superinduced a constitutional septic condition, and would only feed the localized peritonitis into a general one.

I have somewhat digressed from the subject in dwelling upon the treatment of this case, for I have not intended to treat upon chronic metritis, or endometritis in any of its forms. The object and scope of this paper is rather to suggest the means of *preventing* these conditions, and I am firmly fixed in my convictions that if *properly* done in every case the per cent. of uterine troubles would be largely decreased, and gynecologists would have less work to do.

In our day, when abortions are so frequent, and when the question how to avoid procreation seems to be the great problem of our social system, this question looms up before us with no ordinary proportions.

In the time allowed me for this paper I could spare but little space to the management of the case after the first operation. Upon the principle that cleanliness is next to godliness, I would feel that this paper was incomplete if I did not drop a word or two of caution on this line. In the subsequent management of the case the syringe is our chief remedy, and yet if not properly looked after it may become one of the most fruitful sources of infection. To use a political figure, the neighborhood syringe is a regular mugwump, and is in every party who may chance to want a douche or an enema. It would not take a microscope to find upon the nozzle of every household syringe several layers of gummy pus, mucous, blood, feces, urine, dirt, etc., etc. Caution to the nurse along these lines will not be sufficient. Personal inspection is the only safeguard, and will pay a large dividend upon the time and attention of the attending physician. The sterilization and straining of the water used as a douche will be time and trouble well spent.

One other caution, and I am done. Do not allow your patient to be in too great a hurry to get up. Two or three days too long is very much better than two or three hours too soon.

In conclusion, I wish to acknowledge my indebtedness to my enterprising young co-partner, Dr. James T. Ross, for much of the enthusiasm which has prompted the indictment of this paper, and for many practical thoughts and suggestions in our private discussions of this subject.

